# Complex Polysaccharide-Based Nanocomposites for Oral Insulin Delivery

**DOI:** 10.3390/md18010055

**Published:** 2020-01-15

**Authors:** Mar Collado-González, Maria Cristina Ferreri, Alessandra R. Freitas, Ana Cláudia Santos, Nuno R. Ferreira, Guzmán Carissimi, Joana A. D. Sequeira, F. Guillermo Díaz Baños, Gloria Villora, Francisco Veiga, Antonio Ribeiro

**Affiliations:** 1 Department of Food Science and Nutrition, University of Leeds, Leeds LS2 9JT, UK; 2 Department of Pharmaceutical technology, Faculty of Pharmacy of the University of Coimbra, 3000-548 Coimbra, Portugal; mariacristina.ferreri01@universitadipavia.it (M.C.F.); alessandra.grintequi@gmail.com (A.R.F.); ana.cl.santos@gmail.com (A.C.S.); candjs21@gmail.com (J.A.D.S.); fveiga@ff.uc.pt (F.V.); 3 Faculty of Pharmacy of the University of Coimbra, 3000-548 Coimbra, Portugal; ricardao@ci.uc.pt; 4 Department of Chemical Engineering, University of Murcia, 30100 Murcia, Spain; guzmancarissimi@gmail.com (G.C.); gvillora@um.es (G.V.); 5 Department of Physical Chemistry, University of Murcia, 30100 Murcia, Spain; fgb@um.es; 6 REQUIMTE/LAQV, Group of Pharmaceutical Technology, Faculty of Pharmacy, University of Coimbra, 3000-548 Coimbra, Portugal

**Keywords:** chitosan, alginate polysaccharide nanocomposite, stability, LUMiSizer

## Abstract

Polyelectrolyte nanocomposites rarely reach a stable state and aggregation often occurs. Here, we report the synthesis of nanocomposites for the oral delivery of insulin composed of alginate, dextran sulfate, poly-(ethylene glycol) 4000, poloxamer 188, chitosan, and bovine serum albumin. The nanocomposites were obtained by Ca^2+^-induced gelation of alginate followed by an electrostatic-interaction process among the polyelectrolytes. Chitosan seemed to be essential for the final size of the nanocomposites and there was an optimal content that led to the synthesis of nanocomposites of 400–600 nm hydrodynamic size. The enhanced stability of the synthesized nanocomposites was assessed with LUMiSizer after synthesis. Nanocomposite stability over time and under variations of ionic strength and pH were assessed with dynamic light scattering. The rounded shapes of nanocomposites were confirmed by scanning electron microscopy. After loading with insulin, analysis by HPLC revealed complete drug release under physiologically simulated conditions.

## 1. Introduction

Marine biopolymers have gained much attention for the development of drug delivery systems. Several mild methods are available for the synthesis of nanocomposites composed of polyelectrolytes [1] that avoid the use of organic solvents [2] and protect the integrity of the polymers and bioactive molecules [3].

The inability to synthesize insulin in the pancreas is the cause of diabetes mellitus [4], the sixth most common cause of death in the world [3]. Insulin administration is indispensable for the treatment of type 1 patients and is required at a later stage in patients with type 2 diabetes [5]. Surprisingly, only 20% of subcutaneously administered insulin reaches its target [6], and oral bioavailability of insulin is even lower at 1%–2% [7,8]. Thus, the development of a drug delivery system that protects insulin from the acidic environment of the digestive system and enzymatic degradation, while enhancing its uptake by the intestinal cells, is a highly sought-after goal [3,9].

**Figure 1:**
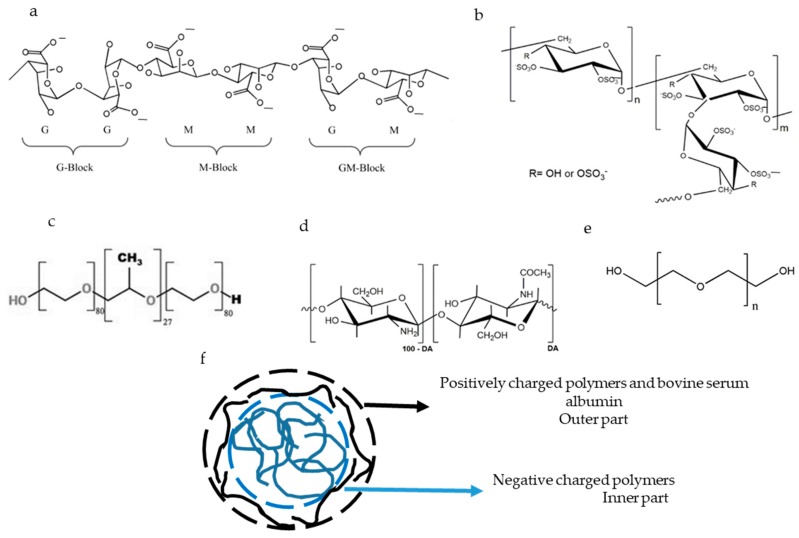
Chemical structure of alginate (**a**) reproduced with permission from Pan et al., Minerals; published by MDPI, 2019 [30], dextran sulfate (**b**) reproduced with permission from Quiñones J.P.; Peniche H. and Peniche C; published by MDPI, 2018 [31], poloxamer 188 (**c**) reproduced with permission from Radacsi et al., Crystal Growth & Design, 2012 [32], chitosan (**d**) reproduced with permission from Quiñones J.P.; Peniche H. and Peniche C; published by MDPI, 2018 [31], and polyethylene glycol 4000 (**e**) reproduced with permission from Zia et al., *Algae Based Polymers*, *Blends*, *and Composites*;published by Elsevier, 2017 [33]. The ideal structure of the nanocomposites is also plotted (**f**).

Chitosan (CS) is a linear polycationic polysaccharide obtained from the deacetylation of chitin (Figure 1). Chitin is a polymer of broad distribution in nature, including marine sources such as shrimp, crab, and squid, among others. CS has been used in oral delivery due to its biocompatibility, non-immunogenicity, nontoxicity, biodegradability, and mucoadhesiveness [3,10,11]. The pKa of this aminopolysaccharide [12] is around 6.5 [8]. Therefore, CS can be used to obtain nanocomposites when interacting with polyanions at a pH below its pKa [4]. In this context, several attempts have been made to develop drug delivery systems consisting of CS and other macromolecules, such as alginate (ALG) [2,13,14], dextran sulfate (DS) [1,11,15,16], poloxamer [17], or acetylated gum [18], or by using crosslinkers such as tri-polyphosphate groups [19]. Typically, the resulting systems are of nanometric size; however, their z potential (ζ) were found to be lower than |25| mV, which is known as the instability region, defining stability as the ability of the nanocomposites to avoid self-aggregation. In water, polyelectrolyte nanocomposites with a ζ higher than +25 mV or lower than −25 mV tend to avoid self-aggregation due to repulsive electrostatic forces [20,21]. In addition to the electrostatic stabilisation, the steric stabilisation of the nanocomposites can be obtained by protein–polysaccharide interactions [22].

ALG is a linear polyanionic polysaccharide isolated from brown marine algae (Figure 1). Chemically, it is composed of mannuronic acid residues (M; pKa = 3.38) which are linked by β-1-4 linkage to its C5-epimer, guluronic acid residues (G; pKa = 3.65) [23]. ALG has been used to develop nanocomposites due to its capacity to form gels after interacting with dicationic ions. A suspension of ALG in a pre-gel state can be stabilised by the addition of a polycationic polymer [3,13,14]. ALG-based nanocomposites have been used for the oral delivery of drugs because they resist the acidic pH of the stomach, protecting the encapsulated drug, and swell in the intestinal pH [6,10,13] where the payload can be delivered. Moreover, ALG favours the loading of insulin in CS-based nanoparticles [24].

DS is a branched polyanion polysaccharide consisting of glucose residues linked by α-1-6 linkage in the main chain and α-1-3 linkage with glucose in ramifications (Figure 1). DS has 2.3 negative charges per monomer [15] and interacts electrostatically with CS [1,16].

Poloxamer 188 is an amphiphilic molecule consisting of a hydrophobic propylene oxide, which is surrounded by two hydrophilic parts of polyethlylene oxide (Figure 1). Poloxamer forms micelles in hydrophilic solvents [25,26,27] and is incorporated in gels through hydrogen bonds [17], stabilising the gels sterically by reducing the aggregation [28].

Poly(ethylene glycol) (PEG) 4000 (Figure 1) acts by stabilising the nanocarrier system and enhancing the penetration of nanoparticles through the mucus layer of the intestine [8].

Bovine serum albumin (BSA) is a protein which prevents aggregation and protects protein loaded in nanostructures from acidic and enzymatic degradation [22,29].

In summary, chitosan, alginate and dextran sulfate are polyelectrolytes used for the formulation and drug delivery systems because they can load and release bioactive molecules. PEG 4000 and poloxamer 188 are non-electrolyte polymers that act in stabilising the structures. BSA is a protein that stabilises the final structure once it is formed.

The potential of chitosan-containing multinanolayers ζ has been shown to depend on pH and ionic strength of the media [34]. Aiming at exploitation of chitosan-containing nanocomposites in oral delivery of insulin, some fundamentals of physicochemical properties of this biopolymer cannot be overlooked. The development of nanocomposites for oral delivery of insulin, which do not aggregate in suspension, is the central objective of this work. We describe the production of nanocomposites with strong resistance to aggregation, using different molecular weight (MW) and different concentrations of CS. Characterisation and stability these nanocomposites are evaluated over ionic strength and pH, and the insulin release behaviour is assessed within simulated gastric and intestinal fluids.

## 2. Results

The main goal of this study was to obtain stable nanocomposites for the oral delivery of insulin. Previously, natural biopolymers have been used extensively for developing drug delivery carriers. Woitiski et al. (2009) [29] developed nanocomposites composed of CS, ALG, poloxamer 188, PEG 4000, DS, and BSA. This system showed a good encapsulation efficiency [29] but low stability, which resulted in aggregation after the synthesis. In this work, we modified the preparation of such nanostructures in terms of three aspects: first was decreasing the pH of the BSA solution to 4.6, the second was removing the buffer phosphate from the synthesis process, and the third change was varying the content and type of CS. The rationale for these changes was based on the fact that electrostatic interactions are mainly responsible for leading to the synthesis of these polyelectrolyte nanocomposites. Polyelectrolytes are characterised by the presence of charges along their structures that determine the inter- and intra-chain interactions. The increase of the ionic strength screens the charges on the polyelectrolytes and the electrostatic interactions and the conformation of the polymers are disrupted.

According to the factors above, the electrostatic nature of the polymers is of utmost importance, as can be seen in Table 1, displaying ζ potential and size distribution of polymeric species implicated in the synthesis of nanocomposites. ALG and DS are polyanionic polymers and CS is a polycationic polymer. It also can be appreciated that although poloxamer and PEG 4000 are not polyelectrolytes, these polymers showed negative and positive natures, respectively. Finally, it can be noted that at pH 5.1, the BSA was almost uncharged and decreased the pH of the suspension to 4.6, whereas the BSA became slightly positively charged and monodisperse (Table 1).

**Figure 2:**
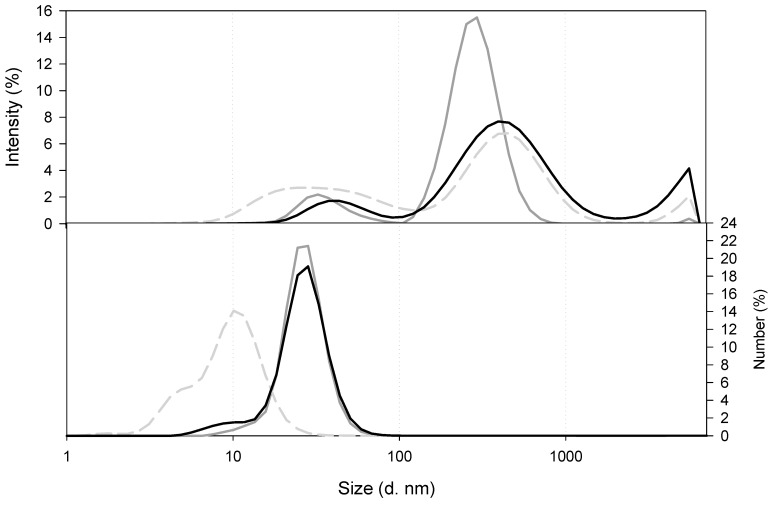
Comparison of the size of CS in solution at pH 4.6. LMW CS 7 × 10^−4^ g/mL (grey dashed line), LMW CS 1 × 10^−5^ g/mL (dark grey solid line), and MMW CS 1 × 10^−5^ g/mL (black line).

Before paying attention to the actual nanocomposites it is important to have information about chitosan solutions. As can be seen in Figure 2, the size distribution for low MW (LMW) CS at 10^−5^ g/mL (dark grey solid line) revealed the presence of two populations, one of them located in 10 s of nanometres of diameter and the second one around a few 100 s of nanometres in hydrodinamic diameter. However, at a higher concentration, namely, 7 × 10^−4^ g/mL, LMW CS (grey dashed line) the populations were enlarged and became larger than one micron. A third population was enlarged around 1000 s of nanometres, although this population was negligible in the size distribution by number. It is important to point out that the size by intensity distribution of LMW CS 7 × 10^−4^ g/mL was quite similar to that of the medium MW (MMW) CS at 10^−5^ g/mL (black solid line). Despite the great differences in the size distribution of LMW CS and MMW CS at 10^−5^ g/mL, both of them had the same moles of amine groups per molecular weight (Table 2). In this work, we used LMW and MMW CS in two different concentrations, namely, 1 × 10^−5^ g/mL and 1 × 10^−7^ g/mL, for preparing nanocomposites, although nanocomposites prepared with LMW CS solution at 7 × 10^−4^ g/mL were used as a reference. Because the volumes of polymer solutions were not changed, the CS content in the nanocomposites was 70 or 7000 times less in nanocomposites prepared with CS solutions at 1 × 10^−5^ g/mL and 1 × 10^−7^ g/mL, respectively.

**Figure 3:**
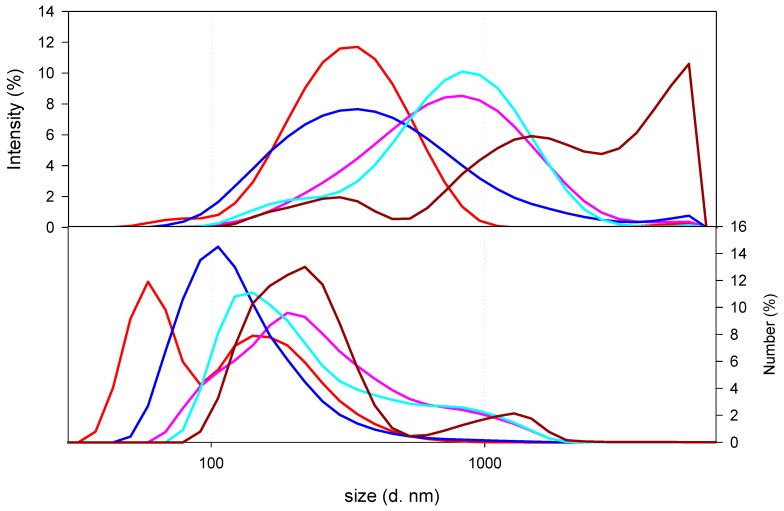
Size of nanocomposites as a function of the type and amount of CS. Nanocomposites prepared with LMW CS solutions at 7 × 10^−4^ g/mL (dark red line), 10^−5^ g/mL (red solid line), or 10^−7^ g/mL (pink line), or MMW CS solutions at 10^−5^ g/mL (blue line) or 10^−7^ g/mL (light blue line) were plotted.

Once the nanocomposites were synthesized, as can be observed in Figure 3, which displays the size distribution of nanocomposites as a function of the type and amount of CS, the size distribution of the nanocomposites prepared with LMW CS 7 × 10^−4^ g/mL solution resulted in three populations: one sized around 100 s of nanometres (300 nm) and the other two having hydrodynamic diameters above 1 micron (namely, 1500 and 4300 nm). When decreasing the concentration of the LMW CS solution used for the synthesis of the nanocomposites, a decrease in the hydrodynamic diameter of the final nanocomposites was observed. As can be seen from Figure 3, the hydrodynamic size of the nanocomposites was dependent on the CS content. Thus, nanocomposites prepared with 1 × 10^−5^ g/mL solution showed lower hydrodynamic size (300 nm) than those prepared with 1 × 10^−7^ g/mL LMW CS solution (900 nm). It is important to point out that similar size distribution was detected when preparing the nanocomposites with MMW CS solutions (500 nm for 1 × 10^−5^ g/mL and 900 nm for 1 × 10^−7^ g/mL). These results reinforce the hypothesis of the existence of electrostatic interactions between the polymers.

Despite the positive charge of the BSA solution at pH 4.6 (Table 1), the adsorption of this protein on the nanocomposites was not enough to change the ζ potential on the nanostructures, as can be appreciated by the fact that all the synthesized nanocomposites were negatively charged (Table 3). These results revealed the presence of negatively charged polymers on the surface of the nanocomposites.

**Figure 4:**
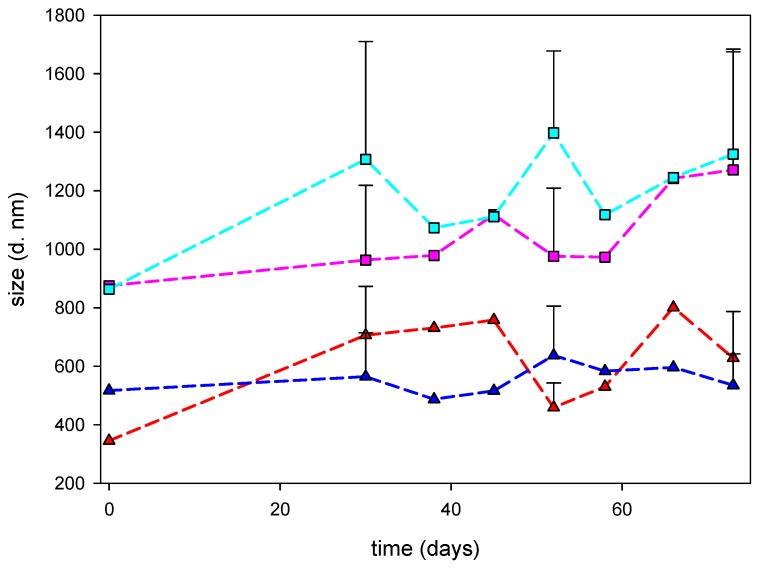
Evolution of the hydrodynamic diameter of nanocomposites after synthesis. Nanocomposites prepared with LMW CS solutions at 10^−5^ g/mL (red line) or 10^−7^ g/mL (pink line) or MMW CS solutions at 10^−5^ g/mL (blue line) or 10^−7^ g/mL (light blue line) were plotted.

The final nanocomposites prepared with CS solutions at 10^−5^ g/mL or 10^−7^ g/mL showed stability in terms of keeping the nanometric size after two months, as the synthesis process was kept at 4 °C and protected from light (Figure 4).

The stability of the nanocomposites immediately after synthesis was also analysed by transmittance profile while centrifuging analyses, performed with LUMiSizer equipment and software [35,36]. This methodology allows for the recording of the migration of the nanocomposites within the suspension while centrifugation is performed. The instability index is a measure of the stability of the suspensions. This parameter is acquired by comparing the transmittance profiles from different suspension samples centrifuged at the same time. During the centrifugation process, the nanocomposites in the suspensions are forced to move due to centrifugal force, which acts on the particles in the suspension. Therefore, the stable equilibrium of forces, which kept the nanocomposites separated, is disrupted. Thus, the lower the instability index is, the more stable the nanocomposite suspension. According to the results displayed in Table 4, the more stable nanocomposites were prepared with MMW CS solution at 10^−5^ g/mL, followed by those prepared with LMW CS solution at the same concentration. On the contrary, the reduction in the CS content, with no regard to its MW, increased the instability of the final nanocomposites. It is important to point out that the hydrodynamic diameters determined for the nanocomposites upon this technique based on particle velocity distribution for sedimentation phenomena were in good agreement with those values obtained by dynamic light scattering.

**Figure 5:**
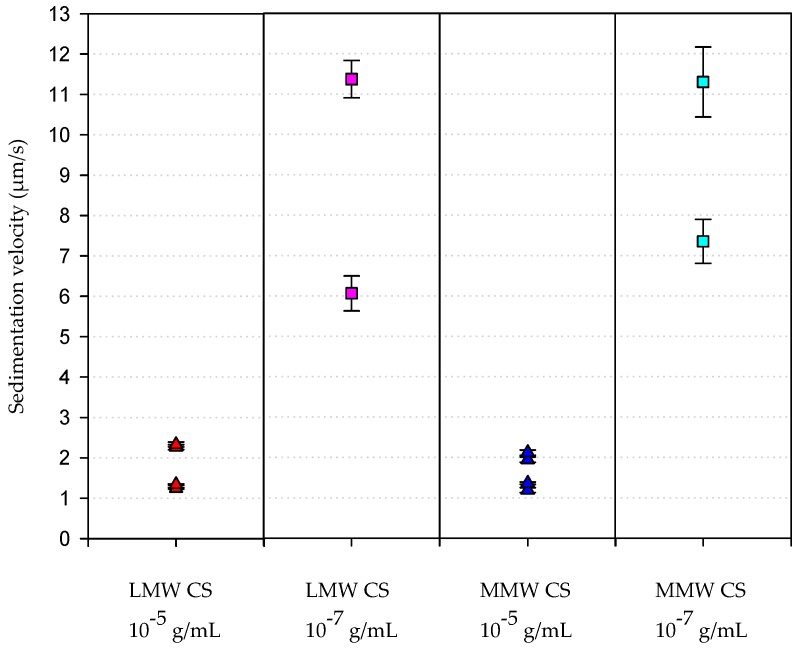
Velocity of sedimentation of the synthesized nanocomposites. This parameter was analysed with LUMiSizer after samples were centrifuged, as indicated in the Materials and Methods section.

The transmittance profiles of the suspension along the time are used to determine the velocity of migration of the nanocomposites in the suspensions. Again, those containing the same amount of CS, with no regard to the MW of the polymer, yielded similar results. Nanocomposites prepared with CS solutions at 10^−5^ g/mL showed the lowest migration velocities, whereas those prepared with CS solutions at 10^−7^ g/mL moved faster in the suspensions (Figure 5).

**Figure 6:**
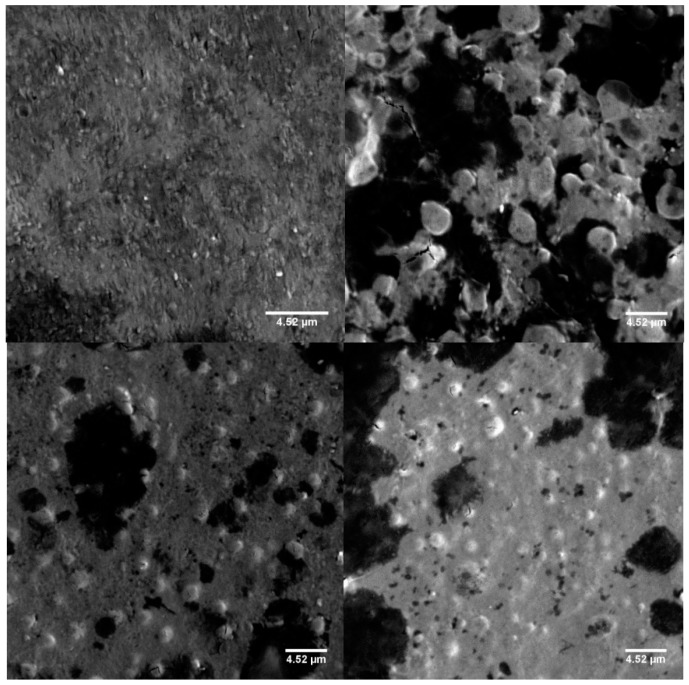
Scanning electron microscope images of nanocomposites prepared with LMW CS solutions at 10^−5^ g/mL (upper left) or at 10^−7^ g/mL (upper right), or MMW CS solutions at 10^−5^ g/mL (lower left) or at 10^−7^ g/mL (lower right). All images were made at a magnification of 2000×. The scale bars in the images represent 4.52 µm.

The scanning electron microscopy (SEM) images (Figure 6) revealed the rounded shape of the nanocomposites synthesised.

The environmental properties of the suspension such as the ionic strength and the pH play an important role in the stability of polyelectrolyte-based nanocomposites [19]. In this work, the effect of the increment of the ionic strength was studied by adding KNO_3_ up to 0.2 M. At this value the ionic strength was higher than that of either simulated gastric fluid (SGF) or simulated intestinal fluid (SIF) to which nanocomposites were exposed. The increase in the ionic strength led to a reduction in the hydrodynamic diameter of the nanocomposites in less than 24 h. According to the results (Table 5), the nanocomposites prepared showed slightly differences in the hydrodynamic size after 24 h of exposure. It is known that when the ionic strength of the medium increases, the polyelectrolytes increase its flexibility and consequently adopt conformations that reduce its expansion in space [37].

Given the interest on the nanocomposites prepared with CS solutions at 1 × 10^−5^ g/mL, the evaluation of the influence of pH was assessed. According to the results, one can notice that nanocomposites upon exposition to pH 1 or pH 7.4 suffered aggregation, as can be detected by the increase in the hydrodynamic size of the structures (Table 6). In some cases, the resulting aggregate was big enough to precipitate or flocculate, and then the clearance of the suspension occurred. This is the explanation for the extreme decrease in the hydrodynamic diameter reported after changing the pH of the suspension.

The HPLC analyses revealed that the insulin entrapment efficiency was close to 100% in all the cases that were studied, as no free insulin was detected after the synthesis of the nanocomposites. The entrapment efficiency was also measured by a direct method; this was found in analysing the release of insulin from nanocomposites in SIF for three hours with shaking. According to the results depicted in the Table 7, and considering that the insulin concentration when preparing the nanocomposites was 78.2 µg/mL, nanocomposites prepared with LMW CS solutions were found to release higher amount of insulin (>90%) compared with those prepared with MMW CS.

**Figure 7:**
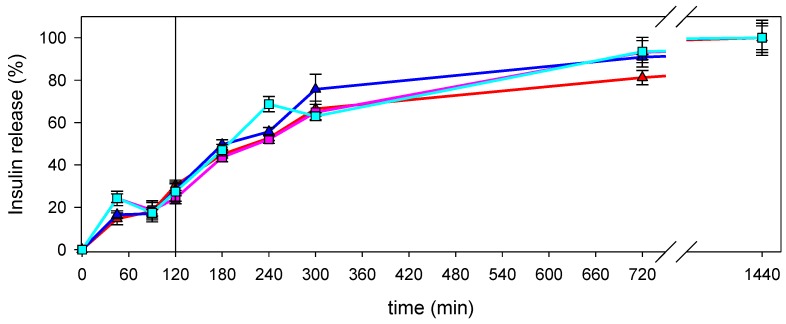
Insulin release from nanocomposites prepared with LMW CS solutions at 10^−5^ g/mL (red line) or 10^−7^ g/mL (pink line), or MMW CS solution at 10^−5^ g/mL (blue line) or 10^−7^ g/mL (light blue line). Vertical line represents the change from simulated gastric fluid (SGF) to simulated intestinal fluid (SIF).

Insulin release under physiological conditions was performed by dialyzing nanocomposite suspensions in SGF for 2 h, which resulted in the release of 30.4% and 24.2% in the case of nanocomposites prepared with LMW CS solutions at 10^−5^ g/mL and 10^−7^ g/mL, respectively, and 28.3% and 27.4% in the case of nanocomposites prepared with MMW CS solutions at 10^−5^ g/mL and 10^−7^ g/mL, respectively. Then, the nanocomposites were transferred into SIF up to 24 h. The cumulative insulin release fitted well with the total amount of insulin used for the formulations (Figure 7).

## 3. Discussion

The formulations of nanocomposites under study involved some polyelectrolytes (ALG, CS, and DS) and other non-polyelectrolyte polymers (poloxamer 188 and PEG 4000). In the synthesis process, polymeric chains with opposite charges interacted through attractive forces entrapping non-polyelectrolyte chains and hydrophilic drugs, such as insulin, which were present in the suspension. Once the core of the nanocomposites was formed, BSA was used for coating in order to protect nanocomposites obtained from the aggregation. Also, electrostatic interactions drove the interaction of the BSA with nanocomposites. Due to the role of the electrostatic interactions, the ζ potential of the polymers in the suspension was of utmost importance and, consequently, was obtained experimentally. It is important to point out that BSA in solution at pH 5.1, as in the standard formulation, had a ζ potential close to 0 mV. Therefore, no electrostatic repulsive forces would appear on the surface of the nanocomposites and the aggregation of the entire structures was promoted, as it has been proposed previously [38]. Nevertheless, the reduction of the pH of BSA suspension to 4.6 resulted in the increase of the ζ potential to +10 ± 3 mV, and thus electrostatic repulsive forces appeared on the surface of the BSA-coated nanocomposites, avoiding the interaction among them. Our results indicate that the BSA changed its conformation when reducing the pH of the solution, as the interaction with the nanocomposites and their resulting stability were different depending on the pH of the solution of the BSA.

Regarding the elimination of phosphate buffer, this change was based on the knowledge that by increasing the ionic strength, the flexibility of the polyelectrolyte changes resulted in changes in their conformation. Moreover, the ionic charges on the polyelectrolytes were screened, resulting in a change in the interactions among molecules. As a result, non-stable nanocomposites were obtained [39,40]. On the contrary, if nanocomposites were synthesised in low ionic strength conditions, being performed without buffer phosphate, the resulting structures were more stable in aqueous suspension. Furthermore, if the ionic strength of the media was increased, the nanocomposites would not aggregate, becoming smaller instead as a result of the change in the flexibility of the polyelectrolytes.

Finally, the concentration of the CS in solution was an important parameter regarding the size of structures in the CS solution. As it has been reported previously [39,41], CS chains become entangled at high concentration and larger structures are obtained. With regard to the effect of CS, we also included CS with different molecular weights (MW) to study the effect of the chain length.

Varying the amount of CS in the final nanocomposites had an impact on their size in the same way as described previously [41]. These authors found a proportion between AuNP/CS or CS/SFN that gave a population with an optimum distribution of sizes of nanocomposites in each system, and an effect of chitosan was claimed. In both cases, nanocomposites were obtained because of the electrostatic interaction between polyelectrolytes. If the proportion of CS or the polyanion in the system was low, large aggregates were obtained. Only a narrow range of proportions yielded nanocomposites with a stable size. Similarly, in this work, those nanocomposites prepared with LMW CS solution at a concentration of 7 × 10^−4^ g/mL yielded nanocomposites, microcomposites, and some millimetric composites, which cannot be detected by dynamic light scattering. By reducing the concentration of LMW CS solution to 10^−7^ g/mL, a broad peak, which spanned from 10 s of nanometers to microns in hydrodynamic diameter, was obtained. However, when the concentration of LMW CS was 10^−5^ g/mL, the nanocomposites obtained were sized in the nanometric range.

The resulting nanocomposites, no matter the amount of CS, showed a negative ζ potential, revealing the presence of CS and PEG 4000 in the inner part of the nanocomposites, in which ALG and DS were likely to be exposed to the surface. The ζ potential of the nanocomposites revealed the electrostatic repulsive interactions among nanocomposites and indicated that the BSA adsorbed on the nanocomposites played a key role in the stabilisation via steric interactions. This contribution of BSA has been previously reported [22,42].

Another property of all the nanocomposites was their stability, defined as no aggregation, at least, in the two months after synthesis, with no variation in the ionic strength or pH, and kept under refrigeration and protected from light.

On the basis of the principles of the centrifugation process and assuming the spherical shape of the nanocomposites in suspension, which could be inferred from the SEM images, the migration velocity could be expressed as in Equation (1):

(1) $$\frac{\omega^{2}r_{\theta }V}{f}\Delta \rho =v_{m}$$

where $\omega^{2}$ is the angular velocity, $r_{\theta }$ is the distance to the rotation axis, V is the volume of the particle, f is the friction coefficient, Δρ is the difference between the density of the particle and the density of the solvent, and $v_{m}$ is the migration velocity. Applying the equations of the friction coefficient and the volume of the sphere, one can obtain Equation (2):

(2) $$kr^{2}=v_{m}$$

where k is a constant and $r^{2}$ is the square of the particle radius. Taking nanocomposites prepared with LMW CS solutions as an example, and considering the hydrodynamic diameters obtained by DLS, one can establish

(3) $$\frac{r_{LMW\;CS\;10^{-5}}^{2}}{r_{LMW\;CS\;10^{-7}}^{2}}=\frac{v_{m\;LMW\;CS\;10^{-5}}}{v_{m\;LMW\;CS\;10^{-7}}},$$

(4) $$0.11=\frac{v_{m\;LMW\;CS\;10^{-5}}}{v_{m\;LMW\;CS\;10^{-7}}}.$$

Thus, Equations (2)–(4) fit well with our experimental results in which the migration velocity of the nanocomposites prepared with CS solution at 10^−7^ g/mL was 10-fold the velocity of those prepared with CS solution of 10^−5^ g/mL. Moreover, it can be assumed that nanocomposite suspensions prepared with CS solution at 10^−7^ g/mL became more heterogeneous after the centrifugation, as can be observed from the difference in the migration velocities of the particles present in the suspension, revealing the presence of populations of nanocomposites showing different hydrodynamic radii. This result revealed that the nanocomposites prepared with CS solutions at 10^−7^ g/mL were indeed more instable.

The increase in the ionic strength of the media resulted in the screening of the electrostatic forces. The screening effect depended on the magnitude of the increment and the exposure of the charges. Increasing the ionic strength up to 0.2 M with KNO_3_ had an impact in the size of nanocomposites similar to that obtained when increasing the ionic strength of the suspensions containing nanocomposites composed of gold nanoparticles (AuNP) and CS (AuNP/CS). In [41], an increase of the ionic strength produced a decrease in the hydrodynamic diameter of the nanocomposites AuNP/CS, indicating that that nanocomposites became more compact.

The strong increase in the hydrodynamic size of nanocomposites while changing medium pH either to 1 or to 7.4 can be related to changes in the conformation of the polyelectrolytes involved in the nanocomposites. At pH 1, polyelectrolytes within the nanocomposites are below their pKa and consequently carboxylic groups in alginate and sulfate in the dextran sulfate are not ionized [43]. Because amine groups in CS are protonated, repulsive electrostatic forces appear among different chains. Therefore, no attractive electrostatic interaction among polyelectrolytes takes part, and hydrogen bonding among chains becomes the main force, determining the gelation of the alginate [44,45], as well as the interaction among PEG and poloxamer with polyelectrolytes. Nevertheless, given that under high ionic strength conditions no aggregation was observed, despite the screening of charges, it is more likely that the denaturation of the BSA, which occurred at pH 2, is the triggering process [46].

The opposite situation was found at pH 7.4. Carboxyl groups in alginate and the sulfate groups in DS are ionized [45]. Amine groups in CS are not ionized and CS becomes non-soluble. Then, repulsive electrostatic forces among ALG and DS predominate resulting in the swelling of the structures. Although BSA structure is not disrupted, the effect of BSA is not enough to maintain nanocomposites as discrete units due to changes occurred in the interactions among polymers within the nanocomposites.

The insulin entrapment efficiency was close to 100%, as it was demonstrated by indirect methods. On the other hand, the direct method for the analysis of entrapment efficiency resulted in the release from 81% to 94%, approximately, of insulin when incubated under SIF for 3 h with shaking. This can be interpreted as the insulin retention within the disrupted nanocomposites.

Under physiological conditions, nanocomposites face a great variety of ionic strength and pH successively. Thus, the way in which the nanocomposites are modified upon those changes cannot always be predictable. This fact can shed some doubts about the effect on the insulin release from nanocomposites. Interestingly, when nanocomposites are incubated under simulated gastric conditions, the insulin is not fully released. This can be interpreted as the nanocomposites acting to protect the insulin, which would be denatured in SGF. Insulin is released upon the exposure to simulated intestinal conditions. Together with insulin protection in gastric conditions, it is one of the objectives pursued when developing oral drug delivery systems for this peptide, that is, to obtain a structure that protects the payload from the effects on the gut and releases the payload in the intestine, where it can be absorbed. Moreover, the CS contained in the nanocomposites could enhance the absorption of the peptide. As it has been previously reported, CS induces the transient opening of paracellular routes [47,48], which allow the absorption of insulin [8,49].

## 4. Materials and Methods

### 4.1. Materials

Low viscosity alginic acid sodium salt from brown algae, which has an M/G ratio of 1.56 [37] (A2158; 100–300 cP, 2% 25 °C, data from the manufacturer); LMW CS (448869-250G) with an MW in the range of 50,000 to 190,000 Da; MMW CS (448877-250G) with an MW in the order of 250,000 Da, with the degree of acetylation being around 20% in both cases (data from manufacturer); and BSA (A1933-25G) were purchased from Sigma-Aldrich Chemie (Schnelldorf, Germany).

Dextran sulfate sodium salt from *Leuconostoc* spp. (5000 Da) and poly(vinylpyrrolidone) K 90 were purchased from Fluka (Buchs, Switzerland); poloxamer 188 (Kolliphor 188) was kindly supplied by BASF (Ludwigshafen , Germany); PEG 4000 was purchased from Fisher Scientific (Leicestershire, UK); calcium chloride was from Riedel-de-Haën (Bucharest, Romania); lactic acid 90% was from VWR BDH Prolabo (Leicestershire, UK); hydrochloric acid 37% and sodium hydroxide were from Merck KGaA (Gernsheim, Germany); and recombinant human insulin 100 IU/mL Actrapid from Novo nordisk A/S (Bagsværd, Denmark).

Stock solutions were prepared in ultrapure water. CS was dissolved in 0.5% (v/v) of lactic acid solution. Solutions were filtered through Millipore #2 filter paper (pore diameter of 0.7 µm) under vacuum. SnakeSkin dialysis Tubing 10,000 MWCO (regenerated cellulose membrane) was purchased from Thermo Fisher Scientific Inc., Waltham, USA. Vivaspin 20 100,000 MWCO polyethersulfone (PES) for ultrafiltration was purchased from Sartorius. Spectra/Por dialysis membranes 100,000 MWCO for the release assays were acquired from Biotech CE Tubing.

### 4.2. Methods

#### 4.2.1. Stock Solutions

In the first step of the nanocomposite synthesis, a solution, that we shall call A, of a final concentration of 6 × 10^−4^ g/mL in alginic sodium salt; 2 × 10^−4^ g/mL DS 5000 Da; and 4 × 10^−4^ g/mL poloxamer 188, and 6 × 10^−5^ g/mL insulin was obtained after stirring for 24 h at room temperature. The pH was adjusted to 4.9 with HCl 0.1 M. A similar solution without insulin was also prepared for the synthesis of blank nanocomposites.

A stock solution of 2 × 10^−3^ g/mL calcium chloride was prepared. 

LMW or MMW CS was dissolved at final concentration equal to 10^−3^ g/mL. Solubility was stimulated by adding 0.5% (*v*/*v*) acid lactic and stirring for 24 h at room temperature. Afterwards, the acidic pH of the solution was increased to 4.6 with NaOH 1 M. The solution was filtered through Millipore #2 paper under vacuum. Then, CS solutions were diluted with MilliQ water to reach a final concentration of 10^−5^ g/mL or 10^−7^ g/mL. After 24 h of stirring, a decrease in pH was observed and the pH was readjusted to 4.6.

For the synthesis of nanocomposites, 3.5 × 10^−3^ g/mL PEG 4000 was dissolved in the solution of CS. After stirring for 24 h, the pH of the solution was readjusted to 4.6 once again. It was observed that, for solutions containing CS at final concentration 10^−5^ g/mL, the pH increased to 4.8. In these cases, the pH was readjusted with no more than one drop of diluted HCl. For solutions containing CS at a final concentration 10^−7^ g/mL, the pH decreased to 3.9, which was readjusted with two drops of diluted NaOH. The solution containing CS and PEG is denominated B.

A 1 × 10^−2^ g/mL BSA solution was obtained after stirring for 4 h at room temperature. Later, the pH was set at 4.6 with HCl 0.1M. 

All solutions were kept at 4 °C until use at room temperature.

#### 4.2.2. Nanocomposite Preparation

Calcium chloride solution (7.5 mL) was added by dropwise extrusion into 117.5 mL of solution A. Then, 25 mL of solution B composed of 10^−5^ g/mL or 10^−7^ g/mL of LMW or MMW CS was added dropwise. Lastly, 25 mL of BSA was added dropwise. Nanocomposite dispersions were concentrated using regenerated cellulose membrane with tubing nominal dry thickness of 10,000 MWCO and a dialysis solution of 10% poly(vinylpyrrolidone) K 90 for 24 h at 4 °C.

#### 4.2.3. Measurements

A Malvern Zetasizer Nano ZS (Malvern Instruments Ltd, Malvern, UK) was used to measure the hydrodynamic size. Measurements were carried out at 25 °C and 173° angle relative to the source. The time autocorrelation function of the scattering intensity fluctuations was used to estimate the hydrodynamic diameter distribution. In the case of CS measurements, each assay consisted of 100 measurements, and in case of nanocomposites, each assay consisted of 6 measurements. In both cases, the number of runs was established by the software in order to reach the quality criteria. Each run lasted 10 s with no delay between measurements. Nanocomposite measurements were carried out after synthesis. Each curve in a plot represented the average of the measurements performed, in a protocol that was confirmed to give very reproducible intensity and number distributions. Distribution by intensity allowed the characterization of particle size, whereas distribution by number, which was obtained by the software assuming the particles to be spherical, the homogeneity of the sample, and the accuracy of the distribution by intensity, allowed the relative populations of the particles to be estimated.

The ζ potential, which is a measure related with the electrical charge in the surface of a particle, was also obtained with the same apparatus. Each assay consisted of three automated measurements.

To study stabilisation after synthesis, the nanocomposite suspensions were kept under refrigeration and protected from light. The measurement of the size of the nanocomposites were performed up to 73 days later, following the indications mentioned above.

To study the stability of the nanocomposites exposed to different pH, the pH of the suspension was set at 1 by adding a solution of HCl or to pH 7.4 by adding a solution of NaOH of the convenient concentration. In order to study the effect of the increase in the ionic strength, KNO_3_ was added to the suspension until a final concentration of the salt equal to 0.2 M. Changes in the hydrodynamic sizes were recorded immediately after the pH or ionic changes and after 2 h and after 24 h. Each analysis consisted of six measurements. Finally, the average and the statistic deviation were calculated for each suspension.

For the determination of the insulin entrapment efficiency, the nanocomposite suspension was ultrafiltered using a Vivaspin 20 100,000 MWCO PES by centrifugation at 6000 *g* for 60 min at 4 °C. The recovered eluent was filtered and diluted 1:2 (v:v) in absolute ethanol and measured in triplicate by high-performance liquid chromatography (HPLC).

Simultaneously, the same suspension of nanocomposites was diluted in a ratio of 1:6 in simulated intestinal fluid (SIF) without pancreatin (USP31-NF26) and stirred vigorously in an orbital shaker for 180 min at room temperature. Then, a sample was diluted 1:2 in absolute ethanol and centrifuged for 10 min at 13,500× *g* at room temperature. After that, the supernatant was filtered using filters with a size pore of 0.22 µm. Finally, the filtrate was assayed in triplicate by HPLC. The insulin entrapment efficiency is the difference between the initial amount of insulin and that measured in the supernatant of the nanocomposites suspension.

For the determination of the insulin release profile in enzyme-free simulated digestive fluids, 15 mL of suspension containing nanoparticles were incubated in 45 mL of simulated gastric fluid (SGF) without pepsin (USP31-NF26) at 37 °C for 120 min under stirring, followed by incubation in 45 mL of SIF without pancreatin (USP31-NF26) for 180 min. Sample aliquots were collected and replaced by the same volume of fresh incubation medium at predetermined times. To determine insulin released from the nanocomposites, samples were diluted at 1:2 in absolute ethanol, centrifuged at 13,500× *g* for 15 min, and the supernatant was assayed for insulin in triplicate by HPLC.

Insulin was assayed using an LC-2010 HT HPLC system (Shymadzu Co., Kyoto, Japan) equipped with a quaternary pump, a UV detector set at 214 nm, a reversed-phase X-Terra RP 18 column 5 µm, 4.6 × 250 mm (Waters Co., Milford, CT, USA), and Purospher STAR RP-18 precolumn 5 µm, 4 × 4 mm (Merck KGa, Darmstadt, Germany). The mobile phase consisted of acetonitrile (A) and 0.1% trifluoroacetic acid (TFA) aqueous solution (B) operated in gradient mode at a flow rate of 1.0 mL min^−1^ set at 30:70 (A/B) changed to 40:60 (A/B) in 5 min for elution during 5 min, and changed to 30:70 (A/B) in 1 min for elution during 1 min. The chromatograms were recorded, and the peak area responses were measured using an automatic integrator. The method was validated and reported to be linear in the range 1.4–112 µg mL^−1^ (*R*^2^ = 0.9998).

Stability over centrifugation was determined by LUMiSizer 611/610 photometric pulsed near-infrared light emitting diods (NIR-LED), at 870 nm (LUM GmbH, Berlin, Germany). LUMiSizer is a new methodology used to test the stability of particles present in a sample. This technique takes advantage of the capacity of the particles in a suspension to reduce the transmittance, which depends on the concentration in terms of mass per volume (g/mL) through the sample in which they are suspended. Combining this feature with the migration of particles in a suspension because of the centrifugation force, the migration of particles along the sample can be seen. Measurements were carried out at 25 °C in rectangular polyamide cell with 10 mm optical path length and polypropylene stopper (110-135XX). Samples were centrifuged at 1056 relative centrifugal acceleration (RCA) until 200 profiles were recorded, taking one every 60 s. In total, 300 profiles were then recorded at 1878 RCA, taking one every 90 s. The instability index is calculated on the basis of the clarification (increase in transmission due to phase separation by sedimentation) at a given separation time, divided by the maximum clarification. The instability index ranges from 0 (more stable) to 1 (more unstable). Relative instability allows comparison of the aggregation and sedimentation speed of nanocomposites, which were centrifuged in the same way. The calculus of the instability index was made by choosing a range in the region of interest in a cell where instability was evident. The analysis time was fixed according to the period of time during which changes in instability were linear for each sample.

The size determination of the particles was performed by using LUMiSizer. For this, analyses were made in three positions of the region of interest in the cell in which the changes in profiles were most pronounced, revealing greater instability of the system. As a result, the software yielded statistical values of the size of the particles of the sample expressed as the harmonic mean in nanometres, and some percentage distributions of size particles in nanometres.

LUMiSizer also allowed the velocity of sedimentation of the particles to be measured in micrometers per seconds (µms^−1^). For this, it was necessary to fix the range in the region of interest in the cell where the most significant changes were detected. Then, a time interval was chosen where particle migration was linear, and the software calculated the velocity of migration of the particles in that region for the time period selected.

## 5. Conclusions

We report the synthesis of nanocomposites with different amounts of two different types of CS. The main objective was to obtain stable nanocomposites for the delivery of insulin. As we have shown, the nanocomposites prepared with a CS solution at 1 × 10^−7^ g/mL, with no regard to the MW, showed larger hydrodynamic size than those prepared with CS solution at 1 × 10^−5^ g/mL, namely, reducing 100 times the CS content resulted in a 2.5-fold increase in size when using LMW CS and 1.7-fold increase when using MMW CS.

All the nanocomposites prepared showed no aggregation after 60 days, which indicates that the formulations obtained were stable for their storage under refrigeration conditions and protected from light. According to the LUMiSizer analyses, the nanocomposites that contained less CS were less stable against centrifugation. The nanocomposites prepared showed good stability against the increment of the ionic strength of the medium, but they collapsed when exposed to pH 1 or 7.4. Interestingly, the HPLC analyses revealed that these nanocomposites were able to protect the insulin from physiological gastric conditions while the peptide was fully released under physiological intestinal conditions, where it could be available for the interaction with absorptive mucosa.

## Figures and Tables

**Table 1:** ζ potential of polymeric species implicated in the synthesis of nanocomposites.

Polymeric Species	pH	ζ Potential (mV)	Relative Abundance (%)	Size Distribution (nm)	Relative Abundance (%)
Alginate	4.9	−52 ± 6 +3 ± 4 −11 ± 3	89.7 7.2 3	385 ± 165 113 ± 33	76.1 23.2
Dextran sulfate	4.9	−54 ± 8 −119 ± 5 −100 ± 4	81.2 5.4 5.2	1220 ± 888 4844 ± 578	90.4 5.8
Poloxamer 188	4.9	−41 ± 6 −22 ± 5	69.9 30.1	638 ± 377 5036 ± 683	95.7 4.3
LMW CS 7 × 10^−4^ g/mL	4.6	+42 ± 8	*	477 ± 256 41 ± 28 4888 ± 769	80.9 16.0 3.2
LMW CS 1 × 10^−5^ g/mL	4.6	+35 ± 6 +8 ± 6	76.8 23.2	298 ± 101 37 ± 14 5463 ± 253	87.5 12.1 0.4
MMW CS 1 × 10^−5^ g/mL	4.6	+35 ± 8 +58 ± 2 +69 ± 8	70.6 11.0 3.1	493 ± 305 4453 ± 1049 46 ± 18	75.6 12.7 11.7
PEG 4000	4.6	+29 ± 7 +5 ± 4	83. 16.7	517 ± 267 4 ± 1 5038 ± 613	80.9 16.0 3.2
BSA	5.1	+2 ± 5	*	25 ± 5 1113 ± 137 8 ± 2	82.5 10.2 5.0
BSA	4.6	+10 ± 3	*	13 ± 2 9 ± 1	72.0 28.0

* When no value is discriminated, the percentage is equal to or higher than 99%. CS: chitosan, LMW: low molecular weight, MMW: medium molecular weight, PEG: poly(ethylene glycol).

**Table 2:** Moles of amine groups in CS solutions, obtained from the degree of acetylation (DA) of CS.

Concentration of CS (g/mL)	Moles of Amine Groups per MW in LMW	Moles of Amine Groups per MW in MMW
1 × 10^−7^	1.2 × 10^−8^	Not used
1 × 10^−5^	1.2 × 10^−6^	1.2 × 10^−6^
7 × 10^−4^	5.7 × 10^−4^	5.7 × 10^−4^

**Table 3:** ζ potential of nanocomposites composed of CS at different concentrations. The results correspond to the average and standard deviation of six measurements.

Formulation	Concentration (g/mL)	ζ Potential (mV)
LMW CS	7 × 10^−4^	−28 ± 9
	1 × 10^−5^	−28 ± 5
	1 × 10^−7^	−30 ± 11
MMW CS	1 × 10^−5^	−31 ± 5
	1 × 10^−7^	−33 ± 10

**Table 4:** Instability index and particle size distribution of the nanocomposites prepared. Analysis was carried out with LUMiSizer. The instability index and span are adimensional parameters. Populations are described by their median (50% in the cumulative analyses) and span (width) of the particle size distribution.

Nanocomposites Prepared with	Instability Index	Median (nm)	Span (×90 to ×10)/×50
LMW CS solution at 10^−5^ g/mL	0.155	485.4	0.7741
LMW CS solution at 10^−7^ g/mL	0.560	857.3	0.7879
MMW CS solution at 10^−5^ g/mL	0.125	465.4	0.7004
MMW CS solution at 10^−7^ g/mL	0.625	924.1	0.7980

**Table 5:** Evolution of the hydrodynamic diameter of nanocomposites exposed to 0.2 M KNO_3_. The hydrodynamic diameter, indicated as z-average, is expressed in nanometres and corresponds to the time before increasing ionic strength (*T*0), just after increasing ionic strength (*T*1), and after 2 h (*T*2) or 24 h (*T*24) of the change.

CS used in the Formulation	Size (d. nm)			
	*T*0	*T*1	*T*2	*T*24
LMW CS 10^−5^ g/mL	478 ± 132	374 ± 60	236 ± 44	271 ± 53
LMW CS 10^−7^ g/mL	896 ± 225	534 ± 137	388 ± 92	311 ± 75
MMW CS 10^−5^ g/mL	600 ± 179	271 ± 52	246 ± 61	269 ± 49
MMW CS 10^−7^ g/mL	1181 ± 335	512 ± 163	341 ± 77	278 ± 57

**Table 6:** Evolution of the hydrodynamic diameter of nanocomposites exposed to pH 1 (top) and pH 7.4 (bottom). The hydrodynamic diameter, indicated as z-average, is expressed in nanometres and corresponds to the time before changing pH (*T*0), just after changing pH (*T*1) and after 2 h (*T*2) of the change.

CS Used in the Formulation	Size (d. nm), pH 1		Size (d. nm), pH 7.4		
	*T*0	*T*1	*T*0	*T*1	*T*2
LMW CS solution at 10^−5^ g/mL	509 ± 121	2 ± 0	520 ± 119	1223 ± 17	1535 ± 93
MMW CS solution at 10^−5^ g/mL	389 ± 73	5404 ± 0	486 ± 141	46 ± 5	48 ± 5

**Table 7:** Insulin release from the nanocomposites prepared with different CS solutions.

CS used in the Formulation	Concentration of Insulin Released	Release (%)
LMW CS solution at 10^−5^ g/mL	73.7 ± 19.0	94.3
LMW CS solution at 10^−7^ g/mL	73.2 ± 17.2	93.6
MMW CS solution at 10^−5^ g/mL	63.9 ± 2.7	81.7
MMW CS solution at 10^−7^ g/mL	63.1 ± 3.8	80.7
